# Computational Analysis of Functional Single Nucleotide Polymorphisms Associated with the CYP11B2 Gene

**DOI:** 10.1371/journal.pone.0104311

**Published:** 2014-08-07

**Authors:** Minyue Jia, Boyun Yang, Zhongyi Li, Huiling Shen, Xiaoxiao Song, Wei Gu

**Affiliations:** 1 Department of Endocrinology and Metabolism, the Second Affiliated Hospital Zhejiang University School of Medicine, Hangzhou, China; 2 Department of Urology, the Second Affiliated Hospital (Binjiang Branch) Zhejiang University School of Medicine, Hangzhou Binjiang Hospital, Hangzhou, China; The University of Hong Kong, Hong Kong

## Abstract

Single nucleotide polymorphisms (SNPs) are the most common type of genetic variations in humans and play a major role in the genetics of human phenotype variation and the genetic basis of human complex diseases. Recently, there is considerable interest in understanding the possible role of the CYP11B2 gene with corticosterone methyl oxidase deficiency, primary aldosteronism, and cardio-cerebro-vascular diseases. Hence, the elucidation of the function and molecular dynamic behavior of CYP11B2 mutations is crucial in current genomics. In this study, we investigated the pathogenic effect of 51 nsSNPs and 26 UTR SNPs in the CYP11B2 gene through computational platforms. Using a combination of SIFT, PolyPhen, I-Mutant Suite, and ConSurf server, four nsSNPs (F487V, V129M, T498A, and V403E) were identified to potentially affect the structure, function, and activity of the CYP11B2 protein. Furthermore, molecular dynamics simulation and structure analyses also confirmed the impact of these nsSNPs on the stability and secondary properties of the CYP11B2 protein. Additionally, utilizing the UTRscan, MirSNP, PolymiRTS and miRNASNP, three SNPs in the 3′UTR region were predicted to exhibit a pattern change in the upstream open reading frames (uORF), and eight microRNA binding sites were found to be highly affected due to 3′UTR SNPs. This cataloguing of deleterious SNPs is essential for narrowing down the number of CYP11B2 mutations to be screened in genetic association studies and for a better understanding of the functional and structural aspects of the CYP11B2 protein.

## Introduction

Single nucleotide polymorphisms (SNPs) are the most abundant class of genetic variations in the human genome with a frequency of approximately every 100 to 300 base pairs [1]. Given that there are millions of SNPs in the entire human genome, SNPs are important as markers for constructing genetic maps and have potential as direct functional variants associated with common and genetically complex diseases and drug responses. The vast majority of SNPs are neutral allelic variants; thus, one of the main goals of SNP research is the identification of functional SNPs, which is a crucial step for understanding the molecular basis of complex traits and diseases in humans [2]. However, the identification of these functional sets of SNPs may be a daunting task. Although experimental techniques will provide the strongest evidence for the functional role of a genetic variant [3], it is not feasible to perform laboratory experiments for all SNPs in the human genome or even in a single gene. Hence, theoretical and/or computational methods are becoming indispensable for the identification and prioritization of SNPs with functional significance from an enormous number of non-risk alleles [4]. Computational methods are sufficiently fast and flexible to provide reliable predictions of functionally significant SNPs with a high accuracy of 80–85% [5]–[9] when combined with sequence, structure, and phylogenetic relationships.

The aldosterone synthase (CYP11B2) gene is situated on chromosome 8q24.3 and encodes aldosterone synthase, which is the key rate-limiting enzyme for the terminal steps of aldosterone biosynthesis [10]. Previously, Strushkevich N and his research group determined the CYP11B2 structure by means of X-ray crystallography [11]. In recent years, there is considerable interest in understanding the possible role of the CYP11B2 gene for assessing the risk associated with corticosterone methyl oxidase deficiency (including CMO I and CMO II), primary aldosteronism, and cardio-cerebro-vascular diseases [12]–[17]. However, most disease association studies have focused on just a few SNPs, particularly T-344C (rs1799998). Other SNPs in the CYP11B2 gene have not been studied, and the *in silico* investigations of SNPs in the CYP11B2 gene remain scarce. Lately, Hui E et al. described a 33-year old Chinese man who was compatible with type 2 aldosterone synthase deficiency carried a heterozygous mutation c.977C > A (p.Thr326Lys) in exon 3 and computational analysis also confirmed the missense variant nocuity [18]. Hence one can see that bioinformatics has its unique advantages in understanding the relationship between genes and diseases. In this study, we performed computational analyses of non-synonymous SNPs (nsSNPs) and UTR-region SNPs in the CYP11B2 gene to identify all of the possible deleterious mutations and propose a modeled structure for the mutant protein. We are confident that the results of our study will provide a further understanding of the CYP11B2 gene in human diseases, as well as a guide for future experimental work.

## Materials and Methods

### Dataset collection

**Figure 1:**
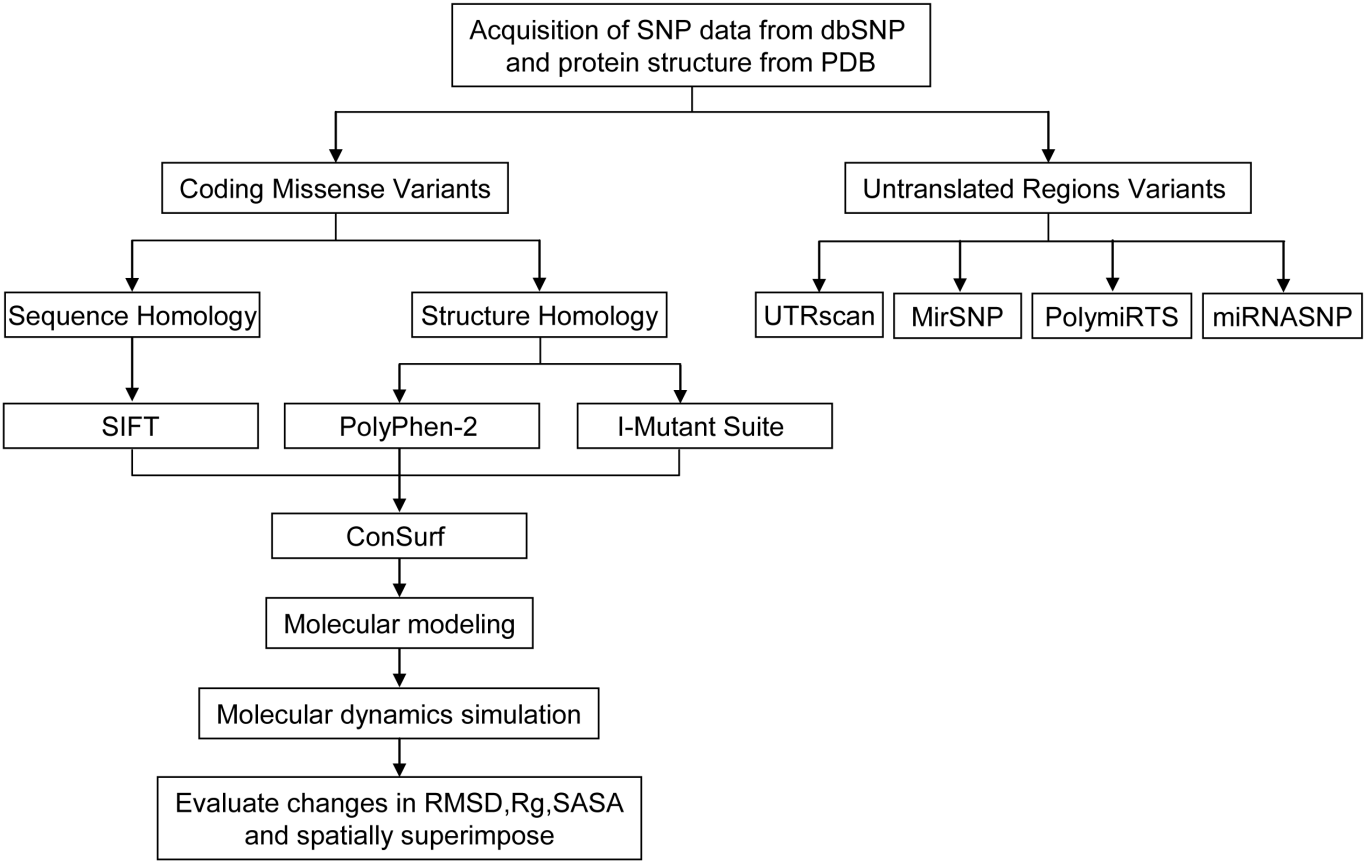
Workflow, tools, and databases used to identify potential functional SNPs in CYP11B2.

The SNP information [SNP ID, amino acid position, mRNA accession number NM_000498.3, and Protein accession number NP_000489.3] of the human CYP11B2 gene used in our computational analyses was retrieved from the National Center for Biotechnology Information (NCBI) database of SNPs (dbSNP (http://www.ncbi.nlm.nih.gov/snp/) [19]. The workflow, tools, and databases used to identify the potential functional SNPs in the human CYP11B2 gene are shown in Figure 1.

### Assessment of nsSNP functionality

The functional context of nsSNPs was predicted using SIFT, PolyPhen and I-Mutant Suite.

SIFT (http://sift.bii.a-star.edu.sg/index.html) is a sequence-homology-based tool to predict whether an amino acid substitution in a protein would be tolerated or damaging [20]. We performed SIFT by submitting the query in the form of SNP IDs or chromosome positions and alleles in nsSNVs tool. Variants at the position with tolerance index score ≤0.05 are considered to be deleterious. A lower tolerance index indicates that the particular amino acid substitution likely has a more functional impact [21], [22].

PolyPhen (http://genetics.bwh.harvard.edu/pph2/) is an automatic tool that predicts the possible impact of an amino acid substitution on a number of features, including the sequence, phylogenetic, and structural information [23]. The query was submitted in the form of protein sequence with mutational position and substitution. The PolyPhen output comprises a score that ranges from 0 to 1, with zero indicating a neutral effect of amino acid substitutions on protein function. Conversely, a high score represents a variant that is more likely to be damaging.

I-Mutant Suite is a suite of support vector machine (SVM)-based predictors of protein stability changes according to Gibbs free energy change, enthalpy change, heat capacity change, and transition temperature [24]. The analyses were performed based on protein sequence combined with mutational position and correlated new residue. And the output result of the predicted free energy change (DDG) classifies the prediction into one of three classes: largely unstable (DDG < −0.5 kcal/mol), largely stable (DDG>0.5 kcal/mol), or neutral (-0.5≤ DDG≤0.5 kcal/mol). I-Mutant Suite is available at http://gpcr2.biocomp.unibo.it/cgi/predictors/I-Mutant3.0/I-Mutant3.0.cgi.

### Evolutionary conservation analysis of nsSNPs

An amino acid that plays an essential role, e.g., in enzymatic catalysis, is likely to remain unaltered despite random evolutionary drift. Hence, the level of evolutionary conservation is often indicative of the importance of the position for maintaining the protein’s structure and/or function. The ConSurf server is a bioinformatics tool for estimating the evolutionary conservation of amino/nucleic acid positions in a protein/DNA/RNA molecule based on the phylogenetic relationships between homologous sequences [25]. After entering the 3D structure of the query protein, the conservation scores are calculated based on the evolutionary relationships among the protein and its homologs [26], [27]. A conservation score between 1 and 4 is considered variable, whereas a score of 5–6 is intermediate, and a score in the range of 7 to 9 indicates conserved. Using the empirical Bayesian method, the accuracy of the conservation score estimation was significantly improved, particularly when a small number of sequences are used for the calculations [26]. ConSurf is available at http://consurftest.tau.ac.il.

### Evaluation of the functional context of SNPs in the UTR region

The 5′and 3′ untranslated regions of eukaryotic mRNAs (UTRs) play crucial roles in the post-transcriptional regulation of gene expression through the modulation of nucleocytoplasmic mRNA transport, translation efficiency, subcellular localization, and message stability [28]–[30]. The functional impacts of UTR SNPs were analyzed using UTRScan [30], MirSNP [31], PolymiRTS [32] and miRNASNP[33].

The program UTRscan looks for UTR functional elements by searching through user submitted sequence data for the patterns defined in the UTRsite collection. And UTRsite is a collection of regulatory elements located in the 5′ and 3′UTRs whose function and structure have been experimentally determined and published. If different sequences for each UTR SNP are found to have different functional patterns, that particular UTR SNP is predicted to have functional significance. The pattern change included two directions by the influence of SNPs at the UTR regions, either from “have pattern” to “no pattern”, or “no pattern” to “have pattern”. UTRscan is available at http://itbtools.ba.itb.cnr.it/utrscan.

MirSNP is a database of SNPs used for the prediction of whether an SNP within the target site would decrease/break or enhance/create a microRNA-mRNA binding site based on information from dbSNP135 and miRBase 18. Its output of single search by entering the gene name includes mirSVR score, the effect of different alleles, the predicted score, conservative information and Start & End & Binding information. Combined with GWAS or eQTL data sets, MirSNP is highly sensitive and covers most experiments confirmed SNPs that affect miRNA function. MirSNP is available at http://cmbi.bjmu.edu.cn/mirsnp.

PolymiRTS is a database of naturally occurring DNA variations in microRNA seed regions and microRNA target sites. Integrated data from CLASH (cross linking, ligation and sequencing of hybrids) experiments, PolymiRTS database provides more complete and accurate microRNA–mRNA interactions. The polymorphic microRNA target sites are assigned into four classes: ‘D’ (the derived allele disrupts a conserved microRNA site), ‘N’ (the derived allele disrupts a nonconserved microRNA site), ‘C’ (the derived allele creates a new microRNA site) and ‘O’ (other cases when the ancestral allele cannot be determined unambiguously). The class ‘C’ may cause abnormal gene repression and class ‘D’ may cause loss of normal repression control. So these two classes of PolymiRTS are most likely to have functional impacts. PolymiRTS is available at http://compbio.uthsc.edu/miRSNP/.

miRNASNP is a database which predicts the effect (loss or gain of function) of SNPs within pre-miRNA, mature miRNA, miRNA target sequences and flanking regions. Using the SNP IDs of the query protein as an input, it produced a list of targets with energy change, SNP-miRNA/target duplexes and gain/loss effect by SNP in miRNA seed or gene 3′UTR. Focused on the prediction of potential effects on miRNA biogenesis and target binding by SNPs through both prediction and experimental validation, miRNASNP is a useful resource to shed light on further experiments. miRNASNP is available at http://www.bioguo.org/miRNASNP/.

### Molecular modeling and molecular dynamics simulation

A structural analysis was performed to evaluate the structural stability of the native and mutant proteins. The crystal structure of the CYP11B2 protein was acquired from PDB [Protein Data Bank; PDB ID = 4DVQ (A chain)] [34]. The Modeller 9.11 package was used to map the mutations on the structure [35]. Furthermore, we used energy minimization and molecular dynamics simulation (MDS) techniques to understand the structural variations in the mutant protein with respect to the native structure using the NAMD 2.6 package [36]. The native and mutant protein structures were solvated in a water sphere using the VMD 1.9.1 package [37]. The cutoff for electrostatic and Van der Waals interactions was 12.0 Å. The temperature was maintained constant at 310K through the use of Langevin dynamics, which provides a means of controlling the kinetic energy of the system with a damping coefficient (gamma) of 1/ps. The energy minimization and molecular dynamics simulations were performed using the CHARMM force field with 5000 iterations and a 1-ns timescale, respectively. The trajectory files were analyzed to obtain the root-mean square deviation (RMSD), radius of gyration (Rg), and solvent-accessible surface area (SASA).

### Statistical analysis

To determine the differences in the RMSD, Rg and SASA value between native and mutant protein structures, statistical analyses were performed with SAS 9.1 software (SAS Institute, Inc., Cary, NC). If quantitative data both fit the normal distribution and homogeneity of variance, Student’s t-test was used to compare the differences between native and mutant group. Otherwise nonparametric Wilcoxon two-sample test was used. The parameters were summarized by medians and interquartile ranges (IQRs). All P-values are two-sided and less than 0.05 was considered a statistically significant difference.

### CYP11B2 database construction

The database at http://203.81.25.54 contains the results obtained from this work. The natural variants listed in the database come from dbSNP. For each nsSNP, we provide predictions of the function effects using SIFT, PolyPhen-2, and I-Mutant Suite. Meanwhile, we also list the UTR SNPs that were predicted to have functional significance by MirSNP, polymiRTS and miRNASNP. In addition, PDB structure files of native and mutant proteins as well as results of molecular dynamics simulation can be downloaded. This database is freely available and will be regularly updated.

## Results

### SNP dataset from dbSNP

**Figure 2:**
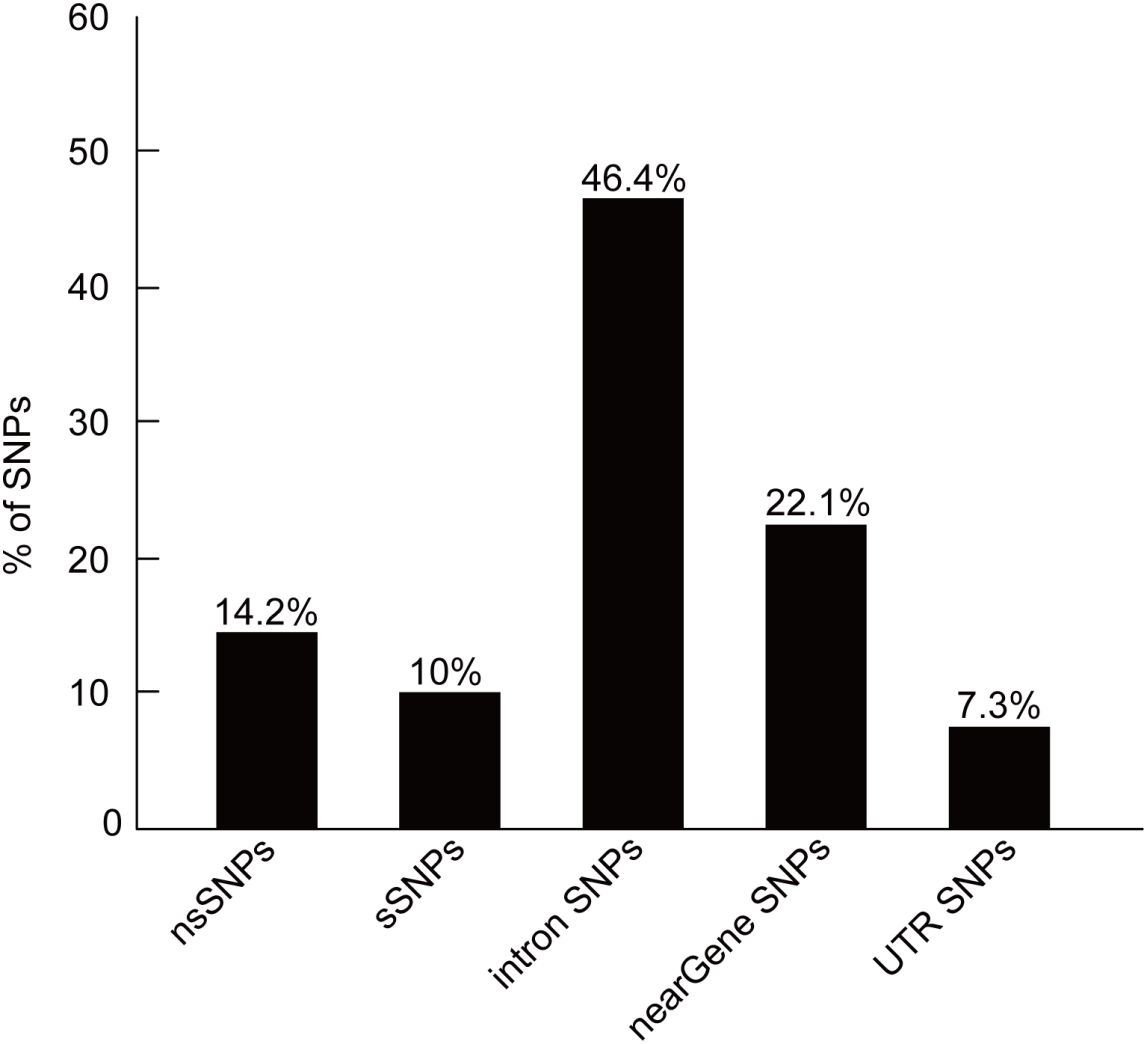
Distribution of SNPs.

The human CYP11B2 gene contains a total of 358 SNPs, of which 51 (14.2%) are nsSNPs and 36 (10.0%) are coding synonymous SNPs. The non-coding region includes 166 SNPs (46.4%) in the intronic region, 79 (22.1%) SNPs in the “near gene” region, and 26 SNPs (7.3%) in the mRNA UTR region. The distribution of SNPs is shown in Figure 2. We selected the nsSNPs and UTR-region SNPs for our subsequent investigations.

### Identification of deleterious and damaging nsSNPs

The identification of the nsSNPs that confer susceptibility or resistance to human diseases should become increasingly feasible with improved *in silico* tools. In this analysis, we employed three *in silico* tools to determine the functional significance of nsSNPs in the CYP11B2 gene. Table 1 presents the results obtained through the SIFT, PolyPhen-2, and I-Mutant Suite analyses of the CYP11B2 nsSNPs.

**Table 1:** Results of SIFT, PolyPhen-2, and I-Mutant Suite.

			SIFT		PolyPhen-2		I-Mutant Suite
dbSNP	Alleles	AA change	Score	Prediction	Score	Prediction	DDG (Kcal/mol)
rs28931609	C/T	R181W	**0.00**	**damaging**	**0.994**	**probably damaging**	−0.19
rs202188379	C/T	A180T	0.16	tolerated	0.014	benign	− **0.52**
rs202173105	C/T	E361K	0.65	tolerated	**0.890**	**possibly damaging**	−0.48
rs201830462	A/G	A320V	0.2	tolerated	0.040	benign	0.42
rs201723054	C/G	A501G	**0.03**	**damaging**	0.286	benign	−**1.43**
rs201485740	C/T	A171T	0.4	tolerated	0.022	benign	−**0.68**
rs201307695	A/C	S387I	0.26	tolerated	0.061	benign	0.08
rs201111434	C/G	E58Q	0.19	tolerated	**0.494**	**possibly damaging**	−0.07
rs200559721	C/T	R22Q	0.12	tolerated	0.003	benign	−0.34
**rs200555543**	**A/C**	**F499C**	**0.00**	**damaging**	**1.000**	**probably damaging**	−**1.78**
rs200283987	C/G	P86A	0.51	tolerated	0.000	benign	−**1.42**
rs199870799	C/T	M92I	0.35	tolerated	0.043	benign	−0.38
rs151052374	A/T	D147E	1.00	tolerated	0.000	benign	−**0.64**
rs150685467	C/G	P418R	0.39	tolerated	**0.601**	**possibly damaging**	−**0.92**
rs150423276	G/T	D317E	**0.01**	**damaging**	**0.971**	**probably damaging**	0.38
rs149963901	A/G	R332W	**0.02**	**damaging**	**0.997**	**probably damaging**	−0.22
rs149902981	A/G	A367V	**0.01**	**damaging**	**1.000**	**probably damaging**	0.3
rs149706111	C/T	V235I	0.11	tolerated	**0.890**	**possibly damaging**	−**0.72**
rs148659506	A/G	R366W	**0.05**	**damaging**	**0.807**	**possibly damaging**	−0.01
**rs147547282**	**C/T**	**Y275C**	**0.00**	**damaging**	**1.000**	**probably damaging**	−**1.04**
rs147109119	A/T	Y61F	1	tolerated	0.000	benign	0.32
rs146765393	G/T	N503T	0.17	tolerated	0.006	benign	0.01
**rs146655862**	**C/T**	**V129M**	**0.00**	**damaging**	**1.000**	**probably damaging**	−**0.88**
rs146241633	C/T	E228G	0.26	tolerated	0.020	benign	−**1.22**
rs145467699	A/G	I248T	0.14	tolerated	0.000	benign	−**1.98**
rs144993866	G/T	L497I	0.25	tolerated	**0.859**	**possibly damaging**	−**1.35**
rs144173527	G/T	Q338P	**0.03**	**damaging**	**0.853**	**possibly damaging**	−0.44
rs144140791	C/T	H225R	0.47	tolerated	0.005	benign	−0.03
rs143027239	A/G	A28V	0.13	tolerated	**0.997**	**probably damaging**	−0.06
rs140991840	C/T	R138H	**0.02**	**damaging**	0.376	benign	−**1.46**
rs140405063	C/T	I339V	0.14	tolerated	0.000	benign	−**0.84**
rs139367087	C/T	Q404R	0.31	tolerated	0.003	benign	−0.37
rs138840536	A/G	T493M	0.08	tolerated	0.036	benign	−0.5
rs138231115	A/C	S315I	**0.04**	**damaging**	**0.992**	**probably damaging**	0.5
rs121912978	C/T	T185I	**0.00**	**damaging**	**0.997**	**probably damaging**	−0.41
rs104894072	A/C	E198D	**0.01**	**damaging**	**1.000**	**probably damaging**	−0.45
rs72554627	T/A	L461Q	0.22	tolerated	0.201	benign	−**1.44**
**rs72554626**	**A/G**	**T498A**	**0.00**	**damaging**	**1.000**	**probably damaging**	−**1.29**
rs61758594	C/G	H63D	0.27	tolerated	0.000	benign	0.05
rs61758593	C/T	M68I	**0.03**	**damaging**	0.002	benign	0.01
rs61757299	C/T	A346T	0.12	tolerated	**0.74**	**possibly damaging**	−0.46
rs61757294	A/G	V386A	0.40	tolerated	0.000	benign	−**2.31**
rs6441	A/G	R30Q	0.59	tolerated	0.000	benign	−**0.62**
rs6438	A/G	A29T	0.36	tolerated	0.001	benign	−**0.73**
**rs5317**	**G/T**	**F487V**	**0.02**	**damaging**	**0.988**	**probably damaging**	−**1.55**
**rs5315**	**A/T**	**V403E**	**0.00**	**damaging**	**0.992**	**probably damaging**	−**1.73**
rs5312	A/T	E383V	**0.01**	**damaging**	**0.741**	**possibly damaging**	−0.01
rs4545	A/G	G435S	0.19	tolerated	0.104	benign	−**1.27**
rs4544	C/T	I339T	0.33	tolerated	0.000	benign	−**1.89**
rs4539	A/G	K173R	0.64	tolerated	0.000	benign	−0.06
rs4537	A/G	N281S	0.53	tolerated	0.000	benign	−0.24

Through SIFT, 19 nsSNPs (37.3%) were predicted to be deleterious with a tolerance score of less than or equal to 0.05. Of these 19 SNPs, seven (R181W, F499C, Y275C, V129M, T185I, T498A, and V403E) were reported to be highly deleterious with a tolerance score of 0.00.

We further analyzed the nsSNPs using PolyPhen based on structural information and multiple sequence alignments. Of the 51 nsSNPs used in our analysis, 14 nsSNPs were predicted to be “probably damaging”, and nine nsSNPs were found to be “possibly damaging”. Consequently, 23 nsSNPs (45.1%) were characterized as damaging.

To improve the prediction accuracy of structure-based tools, we then used I-Mutant Suite. We found that 24 nsSNPs (47.1%) exhibit a DDG value of less than −0.5, which indicates that these are largely unstable.

**Figure 3:**
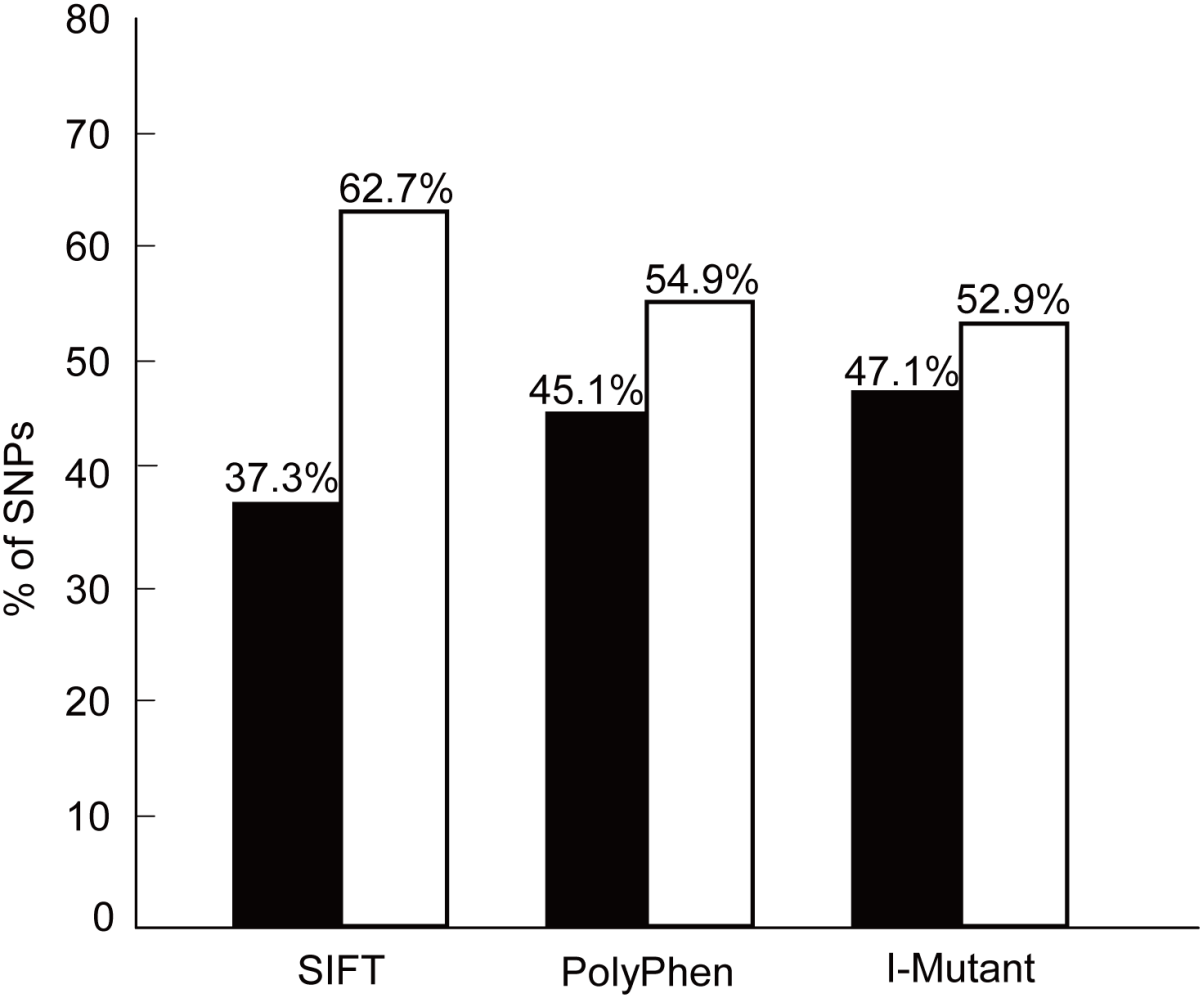
Distribution of deleterious and benign nsSNPs by SIFT, PolyPhen, and I-Mutant Suite. The black rectangular bar indicates the percentage of nsSNPs that were found to be deleterious by SIFT, damaging (Possibly/Probably) by PolyPhen, and largely unstable by I-Mutant Suite. The white rectangle indicates the percentage of nsSNPs that were found to be tolerated by SIFT, benign by PolyPhen, and largely stable/neutral by I-Mutant Suite.

The predictive power of determining the functional impact of a given nsSNP can be significantly increased by combining information from a variety of tools [38]. Accordingly, we combined the SIFT, PolyPhen, and I-Mutant Suite programs to predict the influence of nsSNPs on protein function and structure. Figure 3 shows the distribution of deleterious and benign nsSNPs obtained using SIFT, PolyPhen, and I-Mutant Suite. Of all of the predictions, 37.3%, 45.1%, and 47.1% were specific found by SIFT, PolyPhen, and I-Mutant Suite, respectively. In addition, six nsSNPs (F499C, Y275C, V129M**,** T498A, F487V, and V403E) were predicted to be functionally significant by all three tools. With a diverse set of alignments and molecular characteristics of each *in silico* tool, the results of three tools were slightly different.

### Analysis of nsSNPs in the conserved region

**Figure 4:**
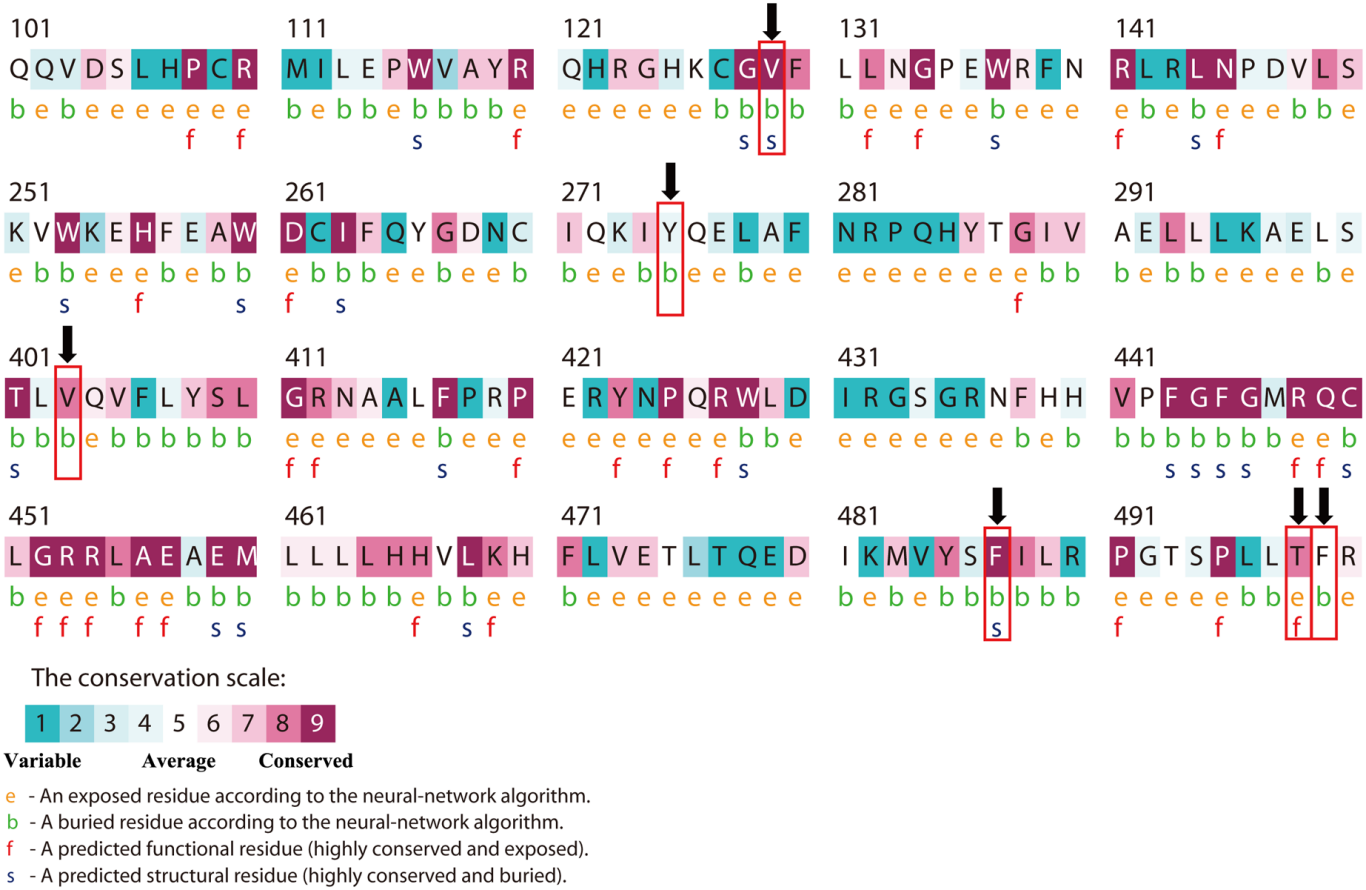
ConSurf output using the UniRef90 protein database. Colors of the ConSurf output indicate the level of sequence conservation. Purple indicates conservation and blue indicates variability. Residues are predicted to be exposed (e), buried (b), functional (i.e., highly conserved and exposed; f), or structural (i.e., highly conserved and buried, s). Numbers indicate residue number of CYP11B2. The bold (black) arrows represent V129M, Y275C, V403E, F487V, T498A and F499C mutation, respectively.

A disease-causing mutation often resides in highly conserved positions. Conservation analyses of the six nsSNPs that were predicted to be deleterious by the above-mentioned three tools were performed using the ConSurf server based on protein structure. Of the six nsSNPs, the four nsSNP positions of V129M, T498A, F487V, and V403E were considered to be located in a highly conserved amino acid region through homologous sequence alignment with the SWISS-PROT, UniProt, and UniRef90 protein databases. The main results are shown in Table 2 and Figure 4.

**Table 2:** Results of the evolutionary conservation analyses using the ConSurf server.

		Conservation score		
dbSNP	Amino acid change	SWISS-PROT	UniProt	UniRef90
rs200555543	F499C	6	5	5
rs147547282	Y275C	5	5	4
**rs146655862**	**V129M**	**9**	**9**	**9**
**rs72554626**	**T498A**	**7**	**8**	**8**
**rs5317**	**F487V**	**7**	**9**	**9**
**rs5315**	**V403E**	**9**	**9**	**8**

A conservation score between 1–4 is considered variable; 5–6 is intermediate; 7–9 is conserved.

### Functional SNPs in the UTR region

UTRs are known to play vital roles in the post-transcriptional regulation of gene expression, and their importance is emphasized by the finding that UTR variations can lead to serious pathology [39]. All of the 26 UTR SNPs were analyzed using UTRscan. After comparing the functional elements for each UTR SNP, we predicted that three SNPs, namely rs61763988, rs35574522, and rs3097, exhibited a pattern change of upstream open reading frame (uORF). Considering the extensive role of UTR SNPs in microRNA binding sites, which could affect the degradation or translational suppression of mRNA, we further analyzed the UTR SNPs by MirSNP, PolymiRTS and miRNASNP. The results showed that 19 SNPs were predicted to change the binding sites with microRNAs by MirSNP and miRNASNP. In PolymiRTS, 11 SNPs were found to highly affect the microRNA binding targets. Then combined the results of these three tools, eight SNPs (rs188784518, rs117910248, rs61763989, rs61757284, rs28390200, rs7463238, rs3802228 and rs3097) indicate a highest likelihood that the polymorphism significantly altered microRNA targeting of the sequence (Table 3).

**Table 3:** The SNPs in the untranslated regions that were predicted to have functional significance by MirSNP, polymiRTS and miRNASNP.

SNPs	Region	Alleles	MirSNP	polymiRTS	miRNASNP
rs188784518	UTR-3	A/C	hsa-miR-664–3p	hsa-miR-664a-3p	hsa-miR-664a-3p
rs117910248	UTR-3	A/G	hsa-miR-711	hsa-miR-711	hsa-miR-711
rs61763989	UTR-3	C/T	hsa-miR-1914–3p	hsa-miR-1914–3p	hsa-miR-1914–3p
			hsa-miR-5194	hsa-miR-5194	hsa-miR-5194
			hsa-miR-423–5p	hsa-miR-423–5p	hsa-miR-423–5p
			hsa-miR-3184–5p	hsa-miR-3184–5p	hsa-miR-3184–5p
				hsa-miR-6738–5p	
				hsa-miR-6762–5p	
				hsa-miR-6845–5p	
rs61757284	UTR-3	A/G	hsa-miR-4432	hsa-miR-4432	hsa-miR-4432
rs28390200	UTR-3	C/T	hsa-miR-5196–3p	hsa-miR-5196–3p	hsa-miR-5196–3p
			hsa-miR-3122		hsa-miR-3122
			hsa-miR-3189–3p		hsa-miR-3189–3p
			hsa-miR-500b		hsa-miR-500b
			hsa-miR-3913–5p		hsa-miR-3913–5p
			hsa-miR-362–5p		
rs7463238	UTR-3	A/G	hsa-miR-1260a	hsa-miR-1260a	hsa-miR-1260a
			hsa-miR-1260b	hsa-miR-1260b	hsa-miR-1260b
			hsa-miR-4758–3p	hsa-miR-4758–3p	hsa-miR-4758–3p
				hsa-miR-3156–3p	hsa-miR-3156–3p
				hsa-miR-188–3p	
				hsa-miR-4258	
				hsa-miR-1224–3p	
				hsa-miR-7108–3p	
rs3802228	UTR-3	A/G	hsa-miR-331–5p	hsa-miR-331–5p	hsa-miR-331–5p
			hsa-miR-4678	hsa-miR-4678	hsa-miR-4678
rs3097	UTR-3	A/G	hsa-miR-4666b	hsa-miR-4666b	hsa-miR-4666b
			hsa-miR-299–3p		

### Molecular dynamics simulation of native and mutant CYP11B2 proteins

To further understand the structural consequences of the prioritized deleterious mutations, molecular dynamics simulations were conducted to analyze the conformational changes in the native and mutant structures (V129M, V403E, F487V, and T498A). The trajectory files were produced after the molecular dynamics simulation, and we then investigated the RMSD, Rg, and SASA variations between the native and the four mutant structures.

**Figure 5:**
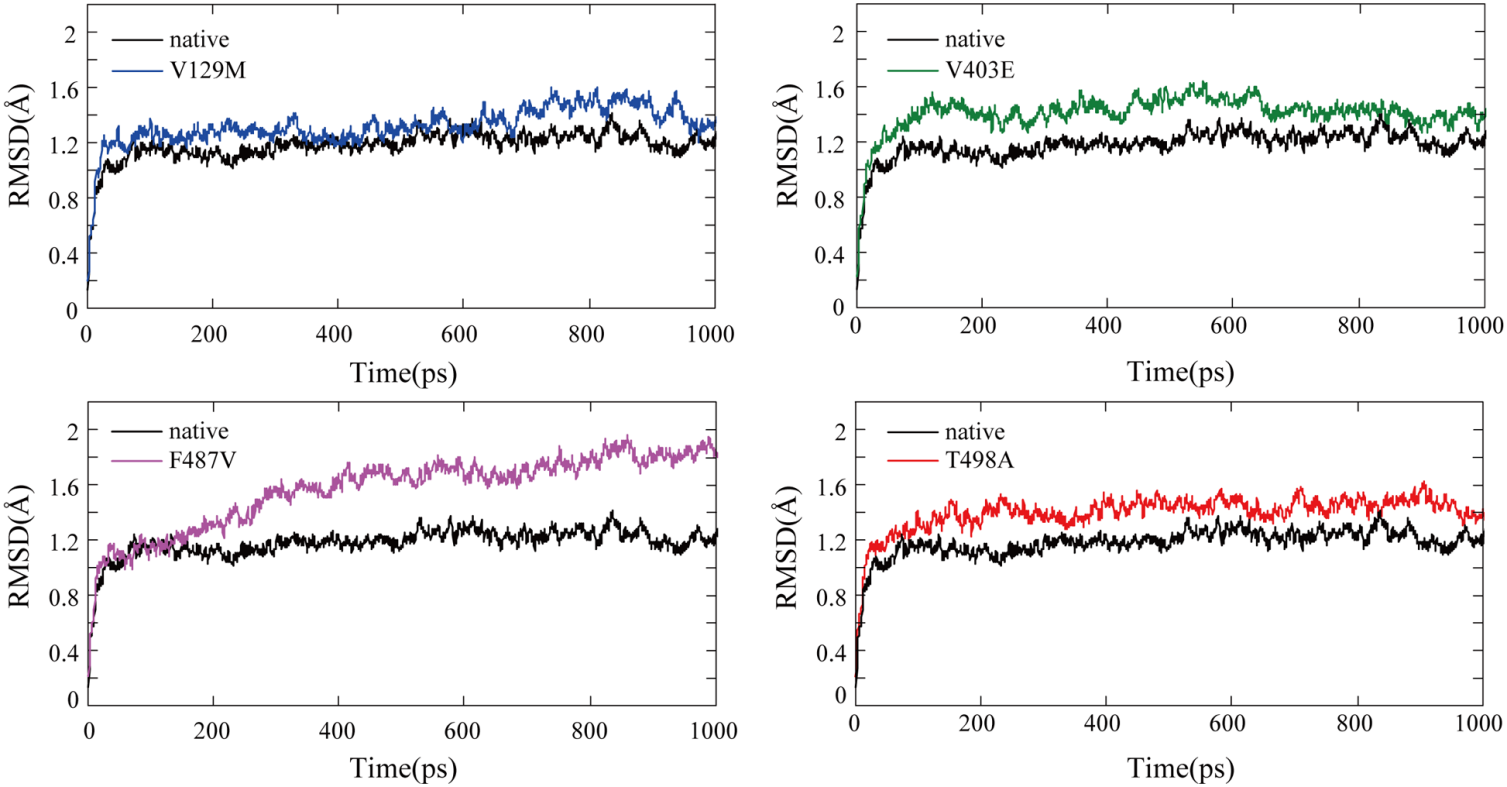
Backbone RMSDs for the native and mutant CYP11B2 protein structures. The ordinate is RMSD (Å), and the abscissa is time (ps). Black, blue, green, violet and red lines indicate native, V129M, V403E, F487V and T498A mutation, respectively.

We calculated the RMSD for all the atoms from the initial structure that was considered as the central origin to measure the convergence of the protein system concerned (Figure 5). In all five structures, considerable structural changes were observed during the initial few picoseconds, leading to an RMSD of ∼1.2 Å and subsequently notable structural deviations during the rest of the simulations. In the last 200 picoseconds of the simulation, the median of RMSD is 1.21(IQR:1.18–1.26) Å for native structure, 1.46(IQR:1.36–1.51) Å for V129M, 1.40(IQR:1.37–1.43) Å for V403E, 1.82(IQR:1.79–1.86) Å for F487V, and 1.47(IQR:1.41–1.50) Å for T498A (Table 4). The statistical analysis showed significant differences between the native structure and the four mutant structures (P<0.0001, particularly F487V). Moreover, small fluctuations in the average RMSD value after the relaxation period led to the conclusion that the simulation generated a stable trajectory and thus provides a credible basis for further analyses.

**Table 4:** Data analyses of last 200 picoseconds of the simulation in RMSD, Rg and SASA.

	native	F487V	V129M	T498A	V403E
RMSD (Å)					
Median(Q1-Q3)	1.21(1.18–1.26)	1.82(1.79–1.86)	1.46(1.36–1.51)	1.47(1.41–1.50)	1.40(1.37–1.43)
P value		<.0001	<.0001	<.0001	<.0001
Rg (Å)					
Median(Q1-Q3)	22.32(22.29–22.35)	22.58(22.55–22.61)	22.40(22.37–22.43)	22.37(22.34–22.39)	22.32(22.29–22.35)
P value		<.0001	<.0001	<.0001	0.8932
SASA (nm^2^)					
Median(Q1-Q3)	24896(24830–24980)	24993(24931–25058)	24821(24753–24895)	24719(24667–24778)	24880(24827–24934)
P value		<.0001	<.0001	<.0001	0.0001

**Figure 6:**
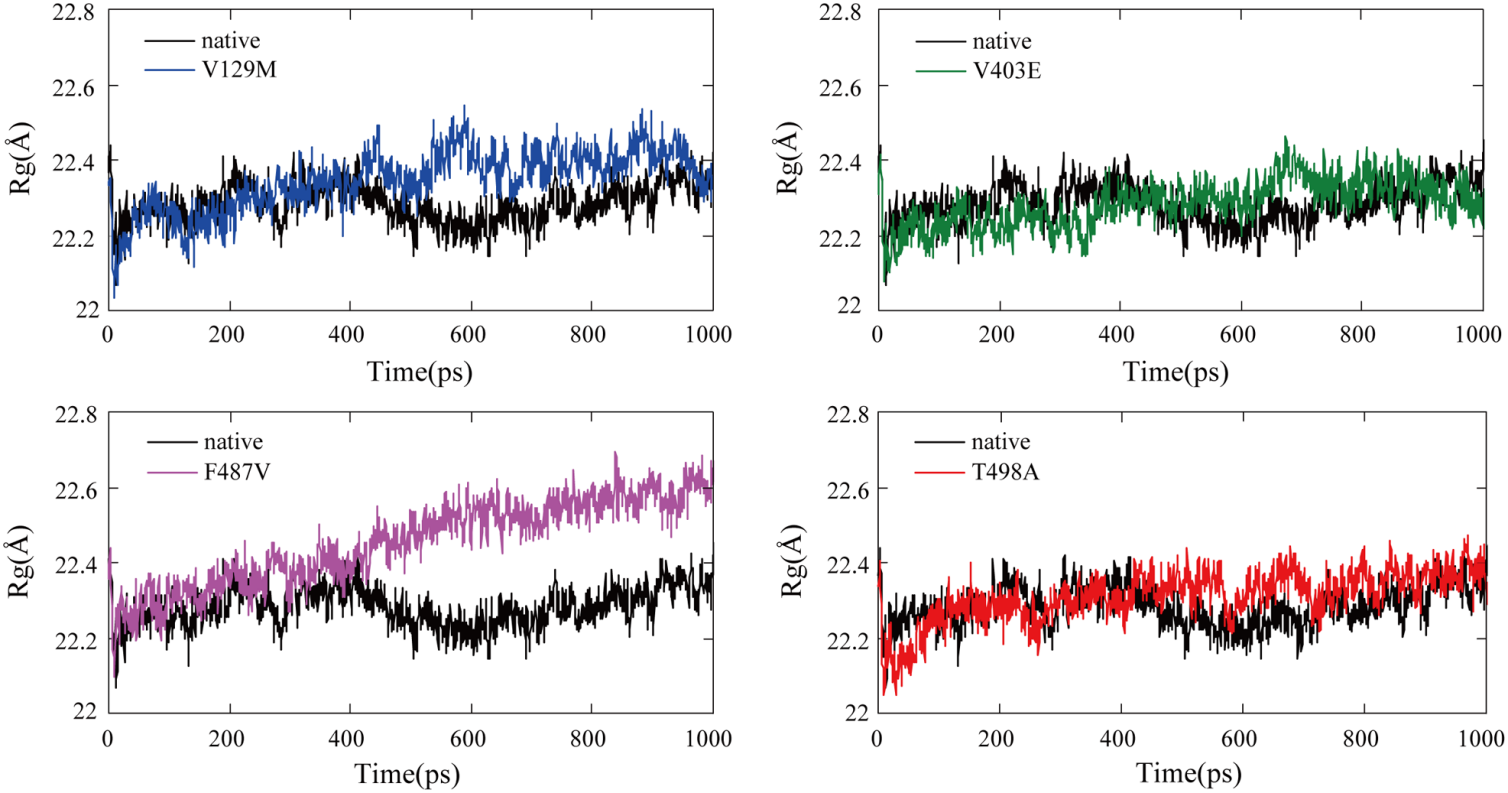
Radius of gyration of Cα atoms of the native and mutant CYP11B2 proteins. The ordinate is Rg (Å), and the abscissa is time (ps). Black, blue, green, violet and red lines indicate native, V129M, V403E, F487V and T498A mutation respectively.

Rg is defined as the mass-weight root mean square distance of a collection of atoms from their common center of mass. Hence, it provides insight into the overall dimension of a protein. The Rg plot for the Cα atoms of the protein as a function of time at 310 K is shown in Figure 6 and results of data analyses are shown in Table 4. The statistic analysis of Rg value of the last 200 picoseconds of the simulation showed that F487V, V129M and T498A had significant differences with native structure [native: 22.32(IQR: 22.29–22.35) Å; V129M: 22.40(IQR: 22.37–22.43) Å; F487V: 22.58(IQR: 22.55–22.61) Å; T498A: 22.37(IQR: 22.34–22.39) Å]. As reflected in Figure 6, the F487V mutant curve differed significantly and fluctuated at a higher rate during the simulation time period, indicating that the mutant conformation is flexible throughout the simulation time and that its structure acquires an expanded conformation compared to the native structure. On the contrary, no difference was found between the native structure and V403E structure.

**Figure 7:**
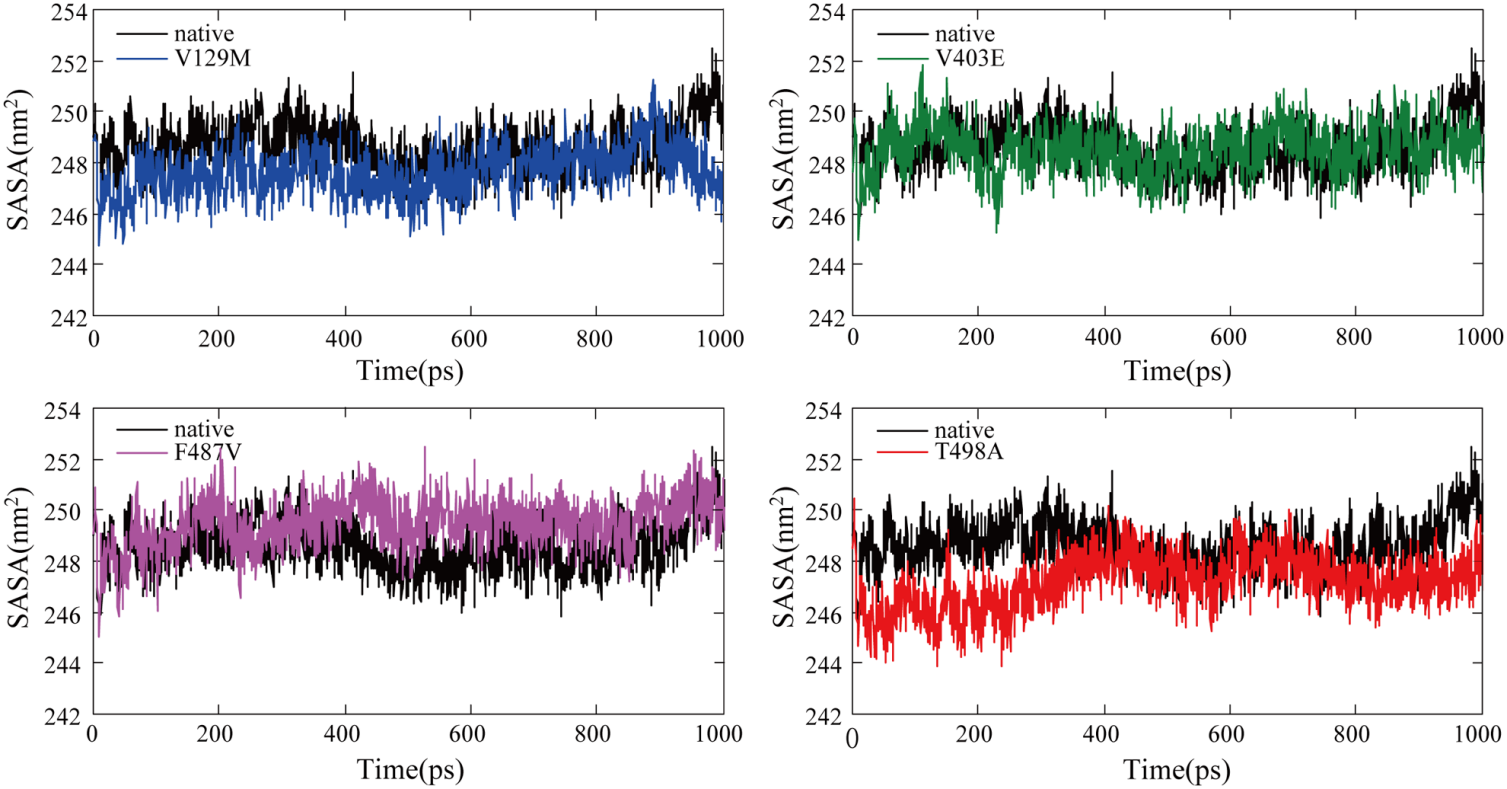
Solvent-accessible surface area (SASA) of the native and mutant CYP11B2 proteins. The ordinate is SASA (nm^2^), and the abscissa is time (ps). Black, blue, green, violet and red lines indicate native, V129M, V403E, F487V and T498A mutation, respectively.

The SASA is the surface area of a biomolecule that is accessible to a solvent and can be related to the hydrophobic core. It is typically calculated using the ‘rolling ball’ algorithm developed by Shrake and Rupley in 1973 [40]. The SASA was calculated for native and mutant trajectories and is depicted in Table 4 and Figure 7. Data analyses showed that there were significant differences between all four mutant structures and native structure [native: 24896(IQR: 24830–24980) nm^2^; V129M: 24821(IQR: 24753–24895) nm^2^; V403E: 24880(IQR: 24827–24934) nm^2^; F487V: 24993(IQR: 24931–25058) nm^2^; T498A: 24719(IQR: 24667–24778) nm^2^]. Compared with the native protein, the F487V mutant protein exhibited a greater value of SASA over time, whereas V129M, V403E and T498A presented lower SASA values. An increase or decrease in SASA indicates changes in the exposed amino acid residues and could affect the tertiary structure of the protein.

**Figure 8:**
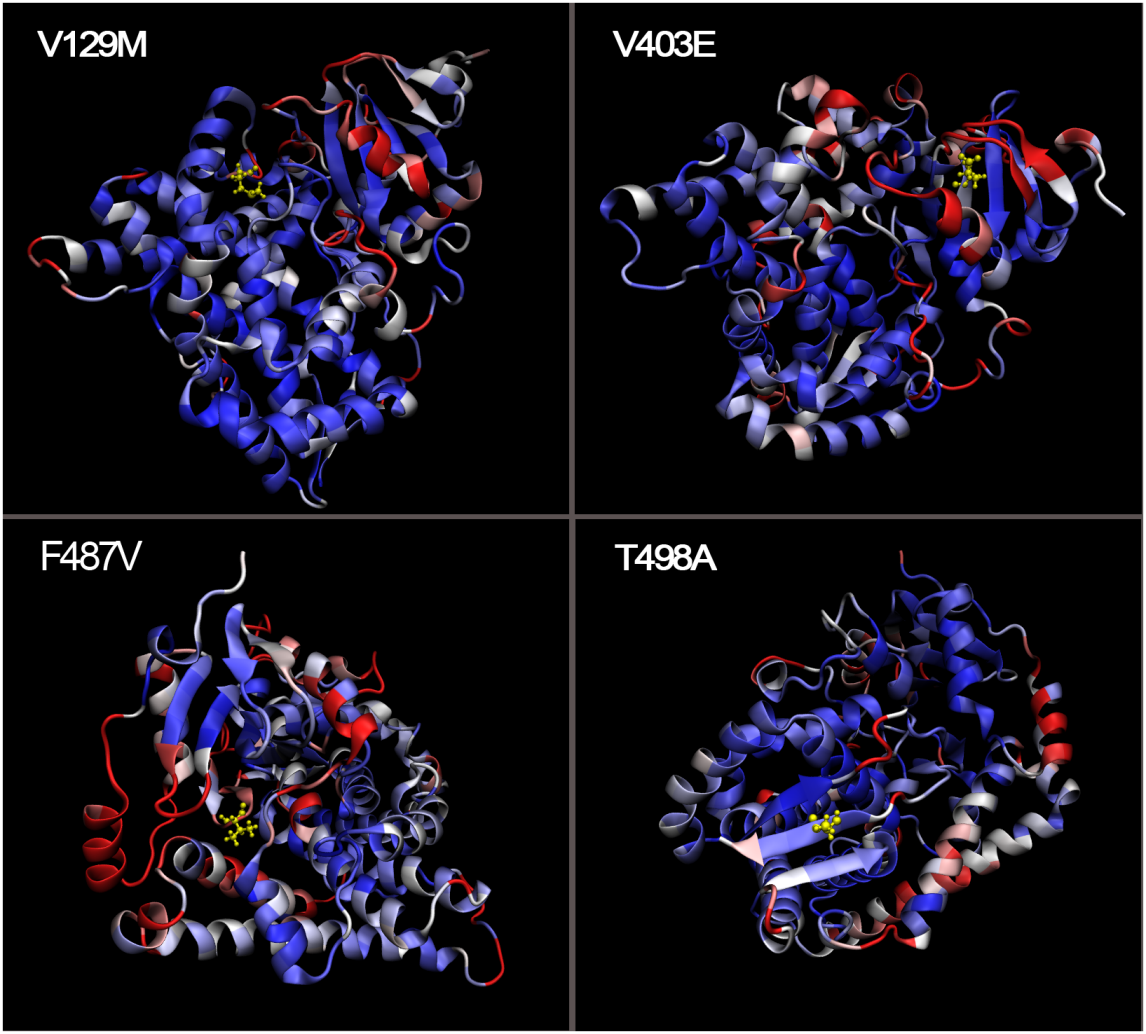
Spatial superimposition of the native and mutant CYP11B2 proteins. Residues with a low displacement (0 Å) are shown in blue, those with a high displacement (5 Å) are shown in red, and those with a moderate displacement are shown in white. The CYP11B2 models are represented in NewCartoon, and the mutated amino acids are represented in CPK.

To properly visualize the crystal structure differences between the native and mutant proteins, we spatially superimposed the molecules (Figure 8). The results show that F487V and V129M exhibit a high displacement (5 Å; shown in red) and that T498A and V403E present a low displacement (0 Å; shown in blue).

Furthermore, we ranked above four SNPs based on results of RMSD, Rg, SASA variations and spatial superimposition (Table 5). So F487V had the highest likelihood of deleterious effect, then V129M, T498A, and V403E with descending perniciousness.

**Table 5:** Ranking SNPs based on molecular dynamics simulation.

dbSNP	AA change	RMSD	Rg	SASA	spatial superimposition
rs5317	F487V	++	++	+	+
rs146655862	V129M	+	+	+	+
rs72554626	T498A	+	+	+	-
rs5315	V403E	+	-	+	-

### CYP11B2 database

During the execution of this project, the CYP11B2 database was created to show a more updated and complete set of *in silico* analyses per mutation. This database allows a user to quickly retrieve and rapidly analyse the predicted effects of protein variants. With its interactive interface, the CYP11B2 database allows dynamic utilization by enabling users to select only the results of the mutations and algorithms that are most important to them. The *in silico* analysis of CYP11B2 in this database will be helpful in the design of further experimental research. The CYP11B2 database is available at http://203.81.25.54/.

## Discussion

Because of the application of high-throughput sequencing technologies, the number of identified genomic variants, particularly SNPs, in the human genome is rapidly growing. The latest release of NCBI dbSNP database (build 141) contains nearly 44 million validated human SNPs [19]. The principal objective of studies in molecular biology and population genetics is to identify and characterize SNPs that are functionally deleterious from neutral SNPs. This is also an inevitable process in genetic association studies of complex genes and diseases [41]. To the best of our knowledge, this study provides the first demonstration of the computational analysis of functional SNPs associated with the CYP11B2 gene. The value and novelty of this study are to prioritize SNPs with functional significance from an enormous number of non-risk alleles and provide new insights for further genetic association studies. Moreover, these identified SNPs could contribute to aldosterone-induced cardiovascular disease, possibly representing novel targets for the therapy. Of 358 SNPs, we selected the nsSNPs and UTR-region SNPs for our investigations, and variants in near-Gene, intronic regions were unexplored.

In this study, we attempted to evaluate the deleterious nsSNPs in three contexts: (1) Identification of deleterious nsSNPs through both sequence- and structure-based methods (SIFT, PolyPhen and I-Mutant Suite), (2) Calculation of the evolutionary conservation of amino acid positions through a conservation score (ConSurf server), and (3) Measurement of alterations in the protein 3D structure due to deleterious nsSNPs through a molecular dynamics approach. Of the 51 nsSNPs associated with the CYP11B2 gene, four nsSNPs, namely F487V, V129M, T498A, and V403E, were finally identified to be highly deleterious based on above comprehensive analyses, particularly F487V.

A number of recent studies mainly focused on the T-344C polymorphism, which impacts the CYP11B2 promoter activity, but the literature on coding substitutions that directly influence the structure of the protein is scarce. However, T498A, one of four above-mentioned nsSNPs that were predicted to be deleterious, was found to be strongly associated with CMO-II deficiency, which shows very low levels of aldosterone synthesis (0.5% or less compared with the wildtype enzyme). The *in vitro* analysis of the enzyme activities of the T498A mutation showed efficient 11 β-hydroxylase activity but a loss of C_18_ activity, resulting in poor aldosterone synthesis [41]. Hence, it appears reasonable to speculate that nsSNPs can ruin the secondary structure of the enzyme, thereby leaving the aldosterone synthase activity intact. It is worth noting that some patients, such as CMO-II deficiency patients who reach adulthood, could be asymptomatic and able to synthesize adequate amounts of aldosterone at the expense of elevated levels of aldosterone precursors. This existence of ostensibly asymptomatic individuals with significantly compromised aldosterone synthase function may reflect problems of ascertainment and may at least partly explain why few coding mutations in the CYP11B2 gene have been reported.

Because the translational regulation of gene expression is as important as the transcriptional regulation for normal cell function and that its dysfunction is related to the pathophysiology of various diseases [42]–[44], the UTR SNPs in the CYP11B2 gene were also evaluated by UTRScan, MirSNP, PolymiRTS and miRNASNP. In our study, we found that 7.3% of the SNPs are located in the UTR region. After comparing the functional elements for each UTR SNP using UTRscan, we found that three SNPs in the 3′UTR were predicted to exhibit a pattern change in their upstream open reading frames (uORFs). However, the uORF in the 3′UTR is hypothesized to have no functional importance.

Due to the importance of the translational regulation of microRNAs, we further studied whether the 3′UTR SNPs change the profile of microRNA binding to the CYP11B2 gene using MirSNP, PolymiRTS and miRNASNP. Of the 26 UTR SNPs, eight (rs188784518, rs117910248, rs61763989, rs61757284, rs28390200, rs7463238, rs3802228 and rs3097) were found to highly affect the microRNA binding targets with MirSNP, PolymiRTS and miRNASNP. These SNPs can break, create, enhance, or decrease microRNA binding (i.e., a single SNP can break a microRNA binding site and also potentially create another site), with consequences on regulation of mRNA degradation pathway thereby affecting mRNA turnover and microRNA function. Therefore, these UTR SNPs could result in the disturbance of aldosterone biosynthesis. Recently, mounting evidence suggests that aldosterone plays crucial roles in a variety of cerebro-, cardiovascular and renal complications [45]. Nevertheless, validation and pathomechanism experiments of these predicted deleterious UTR SNPs were still few. Several studies indicated that rs3802228 might be associated with atrial structural remodeling and the presence of coronary artery disease[46], [47]. As reflected in Table 3, rs3802228 could disturb the interactions between mRNA and microRNA-331–5p. Consistent with this idea, one recent study comes to demonstrate that the upregulation of rno-miR-331* could be seen as biomarkers of prognosis in clinical therapy of heart failure [48]. Besides, rs3097 (G5937C), one of above eight detrimental SNPs, was also found to be associated with cardiac wall thickness [49]. Collectively, these facts and speculations suggest that a potential role of these identified UTR SNPs in the pathogenesis of aldosterone-induced cardiovascular complications. Then, it is of considerable interest that the pathogeny of some cardiovascular disease but not limited to primary aldosteronism could be the variants in the CYP11B2 gene, and aldosterone may act as a central player in this pathological process. Thereby, aldosterone antagonist treatment seems to be of considerable therapeutic value to control and limit the progression of these diseases. This newly pathway of CYP11B2 SNPs/aldosterone/cardiovascular disease opens new research insights and therapeutic avenues for the cardiovascular diseases.

CYP11B2 protein is a steroid hydroxylase cytochrome P450 enzyme involved in the biosynthesis of the mineralocorticoid aldosterone. It is the sole enzyme capable of synthesizing aldosterone in humans and plays an important role in electrolyte balance and blood pressure. Mutations in the CYP11B2 gene can disturb the biosynthesis of aldosterone, then resulting in aldosterone synthase deficiency, also known as corticosterone methyloxidase deficiency. Besides, CYP11B2 gene variations can also change the gene expression, therefore play an important role in many diseases, such as hypertension, primary aldosteronism and heart failure. In addition, Nicod *et al*. found that CYP11B2 is also strongly associated with the rate of decline in renal allograft function [50]. Our *in silico* studies identified various deleterious SNPs, and majority of them have not been reported experimentally so far. However, these findings highlight an attractive screening target for disease association studies involved in CYP11B2 protein, and also provide a guide for future experimental work.

Although the prediction of deleterious SNPs seems to be more and more accurate when integrating more valuable informations, there still exist some challenges to deal with. Computational tools can predict a variant is deleterious or not with a strong confidence, but the information about which disease the variant is related to and which disease the variant has a casual relation with is still missing [51]. In addition, facts show that variants in regulatory regions may alter the consensus of transcription factor binding sites or promoter elements; variants in the introns and silent variants in exons may alter splicing efficiency. Nevertheless, prediction of these variants from genomic sequence remains one of the most challenging tasks for bioinformatics. The biggest problem is over-prediction: (1) the prediction of promoter was expressed cryptically; (2) the vast majority of transcription factor binding sites lack characteristics either in length or sequence; (3) *cis*-regulatory elements, such as ESE (exonic splicing enhancers), ESS (exonic splicing silencers), ISE (intronic splicing enhancers) and ISS (intronic splicing silencers) sites are very poorly defined and may be located in almost any position within exons and introns. For these reasons, we currently did not perform the prediction of variants in near-Gene, intronic regions.

In summary, using combinational *in silico* investigations, the current study identified four nsSNPs, denoted F487V, V129M, T498A, and V403E, as deleterious to the structure and function of the CYP11B2 gene. The molecular dynamics simulation analyses also confirmed that the four nsSNPs that were predicted to be deleterious may induce changes in the stability of the protein by altering the RMSD, Rg, and SASA. In addition, three SNPs in the 3′UTR were predicted to influence the translation pattern of the CYP11B2 gene through UTRscan analysis, and eight 3′UTR SNPs may affect microRNA binding sites, as determined through MirSNP, PolymiRTS and miRNASNP analyses. Altered CYP11B2 function due to mutations and protein expression may play a critical role in determining susceptibility to complex diseases. This cataloguing of deleterious SNPs is essential for narrowing down the number of CYP11B2 mutations to be screened in genetic association studies and for a better understanding of the functional and structural aspects of the CYP11B2 protein.

## References

[1] Ke X, Taylor MS, Cardon LR (2008) Singleton SNPs in the human genome and implications for genome-wide association studies. Eur J Hum Genet 16: 506–515. 18197193 10.1038/sj.ejhg.5201987

[2] Shastry BS (2002) SNP alleles in human disease and evolution. J Hum Genet 47: 561–566. 12436191 10.1007/s100380200086

[3] Chen X, Sullivan PF (2003) Single nucleotide polymorphism genotyping: biochemistry, protocol, cost and throughput. Pharmacogenomics J 3: 77–96. 12746733 10.1038/sj.tpj.6500167

[4] Xu H, Gregory SG, Hauser ER, Stenger JE, Pericak-Vance MA, et al (2005) SNPselector: a web tool for selecting SNPs for genetic association studies. Bioinformatics 21: 4181–4186. 16179360 10.1093/bioinformatics/bti682 PMC1361283

[5] Chasman D, Adams RM (2001) Predicting the functional consequences of non-synonymous single nucleotide polymorphisms: structure-based assessment of amino acid variation. J Mol Biol 307: 683–706. 11254390 10.1006/jmbi.2001.4510

[6] Ferrer-Costa C, Orozco M, de la Cruz X (2004) Sequence-based prediction of pathological mutations. Proteins 57: 811–819. 15390262 10.1002/prot.20252

[7] Jordan DM, Ramensky VE, Sunyaev SR (2010) Human allelic variation: perspective from protein function, structure, and evolution. Curr Opin Struct Biol 20: 342–350. 20399638 10.1016/j.sbi.2010.03.006 PMC2921592

[8] Saunders CT, Baker D (2002) Evaluation of structural and evolutionary contributions to deleterious mutation prediction. J Mol Biol 322: 891–901. 12270722 10.1016/s0022-2836(02)00813-6

[9] Yue P, Li Z, Moult J (2005) Loss of protein structure stability as a major causative factor in monogenic disease. J Mol Biol 353: 459–473. 16169011 10.1016/j.jmb.2005.08.020

[10] Brand E, Chatelain N, Mulatero P, Fery I, Curnow K, et al (1998) Structural analysis and evaluation of the aldosterone synthase gene in hypertension. Hypertension 32: 198–204. 9719043 10.1161/01.hyp.32.2.198

[11] Strushkevich N, Gilep AA, Shen L, Arrowsmith CH, Edwards AM, et al (2013) Structural insights into aldosterone synthase substrate specificity and targeted inhibition. Mol Endocrinol 27: 315–324. 23322723 10.1210/me.2012-1287 PMC5417327

[12] Kayes-Wandover KM, Schindler RE, Taylor HC, White PC (2001) Type 1 aldosterone synthase deficiency presenting in a middle-aged man. J Clin Endocrinol Metab 86: 1008–1012. 11238478 10.1210/jcem.86.3.7326

[13] Pascoe L, Curnow KM, Slutsker L, Rosler A, White PC (1992) Mutations in the human CYP11B2 (aldosterone synthase) gene causing corticosterone methyloxidase II deficiency. Proc Natl Acad Sci U S A 89: 4996–5000. 1594605 10.1073/pnas.89.11.4996 PMC49215

[14] Jia M, Zhang H, Song X, Pang X, Ye W, et al (2013) Association of CYP11B2 polymorphisms with susceptibility to primary aldosteronism: a meta-analysis. Endocr J 60: 861–870. 23535359 10.1507/endocrj.ej12-0455

[15] Tousoulis D, Androulakis E, Papageorgiou N, Miliou A, Chatzistamatiou E, et al (2013) Genetic predisposition to left ventricular hypertrophy and the potential involvement of cystatin-C in untreated hypertension. Am J Hypertens 26: 683–690. 23479071 10.1093/ajh/hps089

[16] Ji P, Jiang L, Zhang S, Cui W, Zhang D, et al (2013) Aldosterone Synthase Gene (CYP11B2) −344C/T Polymorphism Contributes to the Risk of Recurrent Cerebral Ischemia. Genet Test Mol Biomarkers 17: 548–552. 23701507 10.1089/gtmb.2013.0026

[17] Androulakis E, Tousoulis D, Papageorgiou N, Miliou A, Chatzistamatiou E, et al (2013) Effects of the C-344T aldosterone synthase gene variant on preclinical vascular alterations in essential hypertension. Int J Cardiol 168: 1605–1606. 23490082 10.1016/j.ijcard.2013.01.035

[18] Hui E, Yeung MC, Cheung PT, Kwan E, Low L, et al (2014) The clinical significance of aldosterone synthase deficiency: report of a novel mutation in the CYP11B2 gene. BMC Endocr Disord 14: 29. 24694176 10.1186/1472-6823-14-29 PMC3976226

[19] Sherry ST, Ward MH, Kholodov M, Baker J, Phan L, et al (2001) dbSNP: the NCBI database of genetic variation. Nucleic Acids Res 29: 308–311. 11125122 10.1093/nar/29.1.308 PMC29783

[20] Kumar P, Henikoff S, Ng PC (2009) Predicting the effects of coding non-synonymous variants on protein function using the SIFT algorithm. Nat Protoc 4: 1073–1081. 19561590 10.1038/nprot.2009.86

[21] Ng PC, Henikoff S (2001) Predicting deleterious amino acid substitutions. Genome Res 11: 863–874. 11337480 10.1101/gr.176601 PMC311071

[22] Ng PC, Henikoff S (2003) SIFT: Predicting amino acid changes that affect protein function. Nucleic Acids Res 31: 3812–3814. 12824425 10.1093/nar/gkg509 PMC168916

[23] Adzhubei IA, Schmidt S, Peshkin L, Ramensky VE, Gerasimova A, et al (2010) A method and server for predicting damaging missense mutations. Nat Methods 7: 248–249. 20354512 10.1038/nmeth0410-248 PMC2855889

[24] Capriotti E, Calabrese R, Casadio R (2006) Predicting the insurgence of human genetic diseases associated to single point protein mutations with support vector machines and evolutionary information. Bioinformatics 22: 2729–2734. 16895930 10.1093/bioinformatics/btl423

[25] Glaser F, Pupko T, Paz I, Bell RE, Bechor-Shental D, et al (2003) ConSurf: identification of functional regions in proteins by surface-mapping of phylogenetic information. Bioinformatics 19: 163–164. 12499312 10.1093/bioinformatics/19.1.163

[26] Mayrose I, Graur D, Ben-Tal N, Pupko T (2004) Comparison of site-specific rate-inference methods for protein sequences: empirical Bayesian methods are superior. Mol Biol Evol 21: 1781–1791. 15201400 10.1093/molbev/msh194

[27] Pupko T, Bell RE, Mayrose I, Glaser F, Ben-Tal N (2002) Rate4Site: an algorithmic tool for the identification of functional regions in proteins by surface mapping of evolutionary determinants within their homologues. Bioinformatics 18 Suppl 1: S71–77. 12169533 10.1093/bioinformatics/18.suppl_1.s71

[28] Mignone F, Gissi C, Liuni S, Pesole G (2002) Untranslated regions of mRNAs. Genome Biol 3: REVIEWS0004. 11897027 10.1186/gb-2002-3-3-reviews0004 PMC139023

[29] Flynt AS, Lai EC (2008) Biological principles of microRNA-mediated regulation: shared themes amid diversity. Nat Rev Genet 9: 831–842. 18852696 10.1038/nrg2455 PMC2729318

[30] Grillo G, Turi A, Licciulli F, Mignone F, Liuni S, et al (2010) UTRdb and UTRsite (RELEASE 2010): a collection of sequences and regulatory motifs of the untranslated regions of eukaryotic mRNAs. Nucleic Acids Res 38: D75–80. 19880380 10.1093/nar/gkp902 PMC2808995

[31] Liu C, Zhang F, Li T, Lu M, Wang L, et al (2012) MirSNP, a database of polymorphisms altering miRNA target sites, identifies miRNA-related SNPs in GWAS SNPs and eQTLs. BMC Genomics 13: 661. 23173617 10.1186/1471-2164-13-661 PMC3582533

[32] Bhattacharya A, Ziebarth JD, Cui Y (2013) PolymiRTS Database 3.0: linking polymorphisms in microRNAs and their target sites with human diseases and biological pathways. Nucleic Acids Research 42: D86–D91. 24163105 10.1093/nar/gkt1028 PMC3965097

[33] Gong J, Tong Y, Zhang H-M, Wang K, Hu T, et al (2012) Genome-wide identification of SNPs in microRNA genes and the SNP effects on microRNA target binding and biogenesis. Human Mutation 33: 254–263. 22045659 10.1002/humu.21641

[34] Strushkevich N, Gilep AA, Shen L, Arrowsmith CH, Edwards AM, et al (2013) Structural insights into aldosterone synthase substrate specificity and targeted inhibition. Mol Endocrinol 27: 315–324. 23322723 10.1210/me.2012-1287 PMC5417327

[35] Eswar N, Webb B, Marti-Renom MA, Madhusudhan MS, Eramian D, et al. (2006) Comparative protein structure modeling using Modeller. Curr Protoc Bioinformatics Chapter 5: Unit 5 6. 10.1002/0471250953.bi0506s15 PMC4186674 18428767

[36] Phillips JC, Braun R, Wang W, Gumbart J, Tajkhorshid E, et al (2005) Scalable molecular dynamics with NAMD. J Comput Chem 26: 1781–1802. 16222654 10.1002/jcc.20289 PMC2486339

[37] Humphrey W, Dalke A, Schulten K (1996) VMD: visual molecular dynamics. J Mol Graph 14: 33–38, 27–38. 8744570 10.1016/0263-7855(96)00018-5

[38] Rajith B, George Priya Doss C (2011) Path to facilitate the prediction of functional amino acid substitutions in red blood cell disorders–a computational approach. PLoS One 6: e24607. 21931771 10.1371/journal.pone.0024607 PMC3172254

[39] Conne B, Stutz A, Vassalli JD (2000) The 3′ untranslated region of messenger RNA: A molecular 'hotspot' for pathology? Nat Med 6: 637–641. 10835679 10.1038/76211

[40] Shrake A, Rupley JA (1973) Environment and exposure to solvent of protein atoms. Lysozyme and insulin. J Mol Biol 79: 351–371. 4760134 10.1016/0022-2836(73)90011-9

[41] Zhu M, Zhao S (2007) Candidate gene identification approach: progress and challenges. Int J Biol Sci 3: 420–427. 17998950 10.7150/ijbs.3.420 PMC2043166

[42] Cazzola M, Skoda RC (2000) Translational pathophysiology: a novel molecular mechanism of human disease. Blood 95: 3280–3288. 10828006

[43] Reynolds PR (2002) In sickness and in health: the importance of translational regulation. Arch Dis Child 86: 322–324. 11970919 10.1136/adc.86.5.322 PMC1751117

[44] Scheper GC, van der Knaap MS, Proud CG (2007) Translation matters: protein synthesis defects in inherited disease. Nat Rev Genet 8: 711–723. 17680008 10.1038/nrg2142

[45] Quinkler M, Born-Frontsberg E, Fourkiotis VG (2010) Comorbidities in primary aldosteronism. Horm Metab Res 42: 429–434. 20049673 10.1055/s-0029-1243257

[46] Huang H, Zhang L, Liu R, Chen YC, Li X, et al (2011) Polymorphisms within micro-RNA-binding sites and risk of coronary artery disease in Chinese: an angiography-based study. Eur Heart J 32: 355–355.

[47] Cao FF, Chen XD, Wang QS, Li L, Wang XF, et al (2009) [Associations of the genetic polymorphisms in CYP11B2 gene with nonfamilial structural atrial fibrillation]. Zhonghua Liu Xing Bing Xue Za Zhi 30: 1069–1072. 20193392

[48] Feng HJ, Ouyang W, Liu JH, Sun YG, Hu R, et al (2014) Global microRNA profiles and signaling pathways in the development of cardiac hypertrophy. Braz J Med Biol Res 0: 0. 10.1590/1414-431X20142937 PMC4075303 24728214

[49] Mayosi BM, Keavney B, Watkins H, Farrall M (2003) Measured haplotype analysis of the aldosterone synthase gene and heart size. Eur J Hum Genet 11: 395–401. 12734545 10.1038/sj.ejhg.5200967

[50] Nicod J, Richard A, Frey FJ, Ferrari P (2002) Recipient RAS gene variants and renal allograft function. Transplantation 73: 960–965. 11923700 10.1097/00007890-200203270-00023

[51] Wu J, Jiang R (2013) Prediction of deleterious nonsynonymous single-nucleotide polymorphism for human diseases. ScientificWorldJournal 2013: 675851. 23431257 10.1155/2013/675851 PMC3572689

